# Whole genome sequence of rhamnolipid synthesizing *Pseudomonas guguanensis* strain SRIHER B649

**DOI:** 10.1128/mra.00874-25

**Published:** 2025-10-16

**Authors:** Rachel Veronica R, Ramyadevi K C, Shailaja V L, Arun Viswanathan, Mary Elizabeth Gnanambal Krishnan

**Affiliations:** 1 Department of Biotechnology, Faculty of Biomedical Sciences and Technology, Sri Ramachandra Institute of Higher Education and Research (SRIHER), Deemed to be University (DU) 204733, Chennai, Tamil Nadu, India; University of Southern California, Los Angeles, California, USA

**Keywords:** *Pseudomonas guguanensis*, WGS, *rhlAB*, rhamnolipid, crude oil pollution, clean water

## Abstract

*Pseudomonas guguanensis* strain SRIHER-B649 was isolated in the oil-contaminated waters of Chennai harbor, India. Whole genome sequencing was performed for the first time to determine the location and size of the rhamnolipid synthesizing *rhlAB* gene. Genetic engineering experiments can undoubtedly benefit from the collected data for this strain.

## ANNOUNCEMENT

Hydrocarbon-degrading organisms were screened at Chennai harbor waters (13.0815°N, 80.2921°E) by serial dilution method, and a bacterium grew well in 2.5% hexadecane-enriched Bushnell-and-Haas medium. It produced a high-molecular-weight monorhamnolipid (1,264.52 Da) (PubChem SID 462764885) and emulsified hexadecane (56%) (Indian Patent Grant 548405) (1, 2, 3) and is now acclimatized to grow at higher hydrocarbon concentration. To characterize the bacterium, DNA extraction was performed (Barker) (4). 16S rRNA was amplified (27-F-AGAGTTTGATCCTGGCTCAG, 1492-R-GGTACCTTGTTACGACTT) (5) and sequenced (ABI-3730 DNA analyzer; Thermo Fisher, USA) and compared with the GenBank data set (NCBI BLAST, v.2.2.12) (6). The bacterium was identified as *Pseudomonas guguanensis* (GenBank accession no. KU302611.1), which was reconfirmed at IMTECH, Chandigarh. Though this bacterium was previously discovered in the Guguan islands, Taiwan (7), there are no reports on hydrocarbon degradation.

It is known that rhamnosyltransferase genes (*rhlAB*) are involved in the biosynthesis of this high-molecular-weight emulsifier. Location and identification of this gene required WGS information because only gene scaffolds were available (GCF_900104265.1) (8). Thus, genomic DNA was extracted from *P. guguanensis* (peptone-yeast medium) following protocols listed previously (9), with the following modifications: (i) CTAB without β-mercaptoethanol, (ii) incubation at 95°C (1 h) instead of 65°C (30 mins), (iii) chloroform instead of chloroform-isoamyl alcohol, and (iv) addition of RNase A (10 mg/mL) at the initial steps of chloroform incubation. DNA fragmentation and library construction were carried out per protocols by Nextera DNA Flex Library Prep Kit (Illumina, USA) using on-bead tagmentation chemistry. After library construction, dual index adapters were ligated at the blunt ends and purified. Quality and quantity of the fragment library were estimated using Agilent (G2964AA) automated electrophoresis (Agilent Technologies, USA) using 2200 TapeStation Software (v.A.01.02) and Qubit dsDNA HS assay kit (Thermo Fisher Scientific, USA), respectively. A good-quality library was sequenced (2 × 250 bp chemistry, Illumina MiSeq platform, USA) at the National Centre for Microbial Resource, Pune. The total number of raw reads was 967,586, with a read length of 250 bp for R1-R2. Raw data quality assessment was done using FastQC Toolkit v0.11.8 (10), and bad-quality data were trimmed using NGS QC Toolkit v2.3.3 (11). Genome assembly was done using SPAdes 3.11.1 assembler (12). The genome contained 60 scaffolds, N50 value of 314,320 bp, size of 5,560,392 bp, and coverage of 120×. Default parameters were used for all software unless specified otherwise. The publicly available genome was annotated (NCBI PGAP, v.6.8) (13) with 5,121 predicted coding genes.

**Fig 1:**
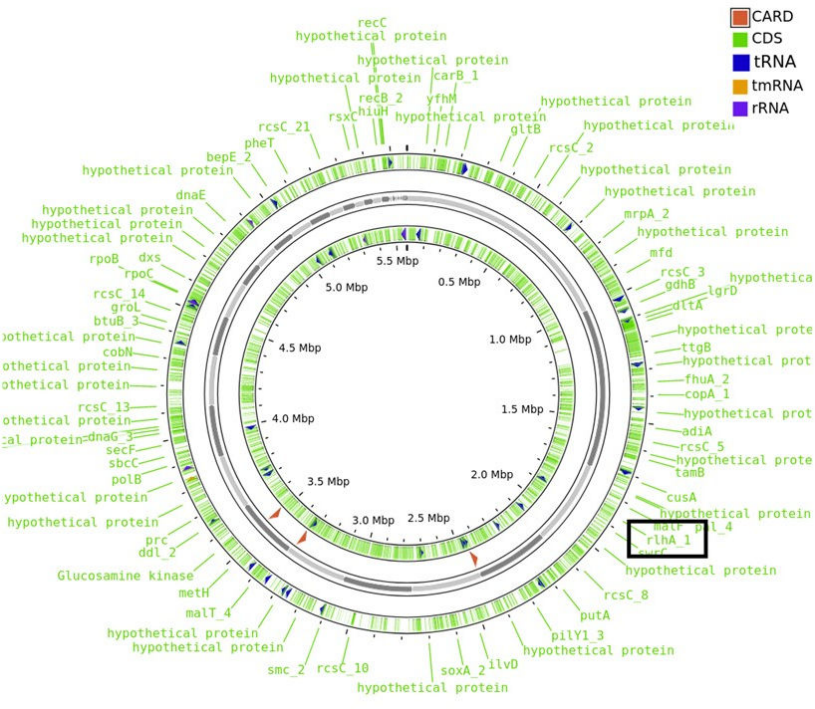
Circular map of *P. guguanensis* genome showing the antibiotic resistance genes (CARD), coding sequences (CDS), transfer RNA (tRNA), transfer messenger RNA (tmRNA), ribosomal RNA (rRNA), and rhlA gene highlighted.

Based on WGS data, the putative *rhlA* gene was identified (Fig. 1, Proksee CGView, v.1.2.0) (14). Since the homology of *P. guguanensis rhlA* was 72.2% with *Pseudomonas aeruginosa* PAO1 (NC_002516.2) and 98.5% with *P. guguanensis* JCM 18416 scaffolds (NZ_FNJJ01000001.1) (NCBI BLAST, v.2.13.0) (6), the following putative *rhlAB* arrangement was deciphered: a 500-bp promoter, an 888-bp putative *rhlA* (as in the WGS datum), a 64-bp short sequence, and a 1,300-bp putative *rhlB,* which is based on the *P. aeruginosa rhl* layout. To ensure that the coding sequences were not omitted, a few nucleotides were flanked to the target gene and thus the total sequence length of *P. guguanensis rhlAB* is 2,752 bp. Thus, the locus and gene sequence of *rhlAB* were identified for the first time in *Pseudomonas guguanensis*, which will be useful for researchers who work on rhamnosyltransferases in this strain.

## Data Availability

The whole genome shotgun sequence of *P. guguanensis* strain SRIHER B649 has been deposited in GenBank under the BioProject no. PRJNA1014258 and the BioSample no. SAMN37322556 with the accession no. NZ_JAVKYQ000000000 and the version no. NZ_JAVKYQ000000000.1. Raw sequence reads are available at the NCBI SRA database under SRR34789558. The *rhlA* sequence of *P. guguanensis* strain SRIHER B649 has been deposited in GenBank with the accession no. PX069939.1.
